# An Evaluation of 7279 Sports Injuries from a Level 1 Trauma Center with a Focus on Gender Differences

**DOI:** 10.3390/jcm11061708

**Published:** 2022-03-19

**Authors:** Maria Alexandra Bernstorff, Johanna Schlombs, Norman Schumann, Thomas Rosteius, Ole Somberg, Maximilian Wenzel, Thomas Armin Schildhauer, Matthias Königshausen

**Affiliations:** 1 Medical Department, University Hospital Bergmannsheil Bochum, Ruhr-University of Bochum, Bürkle de la Camp-Platz 1, 44789 Bochum, Germany; thomas.rosteius@bergmannsheil.de (T.R.); ole.somberg@bergmannsheil.de (O.S.); maximilian.wenzel@bergmannsheil.de (M.W.); thomas.schildhauer@bergmannsheil.de (T.A.S.); matthias.konigshausen@gmail.com (M.K.); 2 Department of Medicine, Ruhr-University Bochum, Universitätsstraße 150, 44801 Bochum, Germany; johanna.schlombs@ruhr-uni-bochum.de; 3 Department of Mathematics, Ruhr-University Bochum, Universitätsstraße 150, 44801 Bochum, Germany; norman.schumann@ruhr-uni-bochum.de

**Keywords:** sports trauma, gender, emergency, sex differences

## Abstract

Purpose: Hardly any other topic is as current as the gender-conscious medical treatment of every individual. Similarly, in sports, there are crucial differences that should be considered in order to treat everyone appropriately, with the aim to prevent injuries according to their needs. The objective of this study is to show that the differences between biological males and females are so significant that they require both specific injury prevention and therapy programs. Methods: This study is a retrospective analysis of letters from a department of orthopedics and trauma surgery between the years 2000 and 2015. Results: The analysis of 20,567 inpatient and outpatient letters of a level 1 trauma center in Germany revealed that 5455 patients suffered 7279 injuries; 1475 of the patients were female and suffered 2035 injuries, and 3890 patients were male and suffered 5244 injuries. Conclusion: The results show the differences between males and females with regard to sport-specific injuries, pointing out the sex differences in the sport-specific area in relation to the body region.

## 1. Introduction

Sports and recreational physical activity are considered to be beneficial to health, and the general population is encouraged to increase their physical activity [1].

In Germany, it has been observed for several years that the percentage of adults who follow these WHO recommendations is steadily increasing [2].

The positive effects that physical sporting activity has on various chronic diseases, such as coronary heart disease or diabetes mellitus, have been studied and proven in the past [3,4,5]. However, in order to draw maximum benefits from these positive effects, it is important to find more targeted prevention and treatment approaches for the 1.5 to 2 million sports injuries annually occurring in Germany [6]. Even though most sports injuries heal without negative consequences, there still is a relevant proportion of them that result in long-term physical limitations [7].

There are a number of prevention programs found in the literature that focus on reducing the risk of injury in individual sports [6]. On the one hand, there are programs that specifically focus on one particular sport, and on the other hand, some focus on a specific body region [6,7]. Most of these programs put their emphasis on male athletes [6,8]. Data on injuries related to sex, age, and type of sport form the basis of such prevention programs. With regard to their anatomy, women and girls differ significantly from men and boys in childhood and adolescence. There are clear differences in the growth phases as well as in degrees of maturity in adolescents. This results in different structures being affected by injuries during athletic stress. Therefore, gender differences must logically be included in prevention and therapy. At this point, there is a lack of required prevention programs for females and their active participation in all sports.

The aim of the present work is to show the different body localizations that can be affected and the extent and types of injuries, comparing both sexes and taking corresponding age groups and sportive activity into account, thus creating a basis for sex-specific preventive and therapeutic treatment.

## 2. Methods

This study is a retrospective analysis of letters from the Department of Orthopedics and Trauma Surgery and the Department of Spinal Cord Injury of our institution from 2000 to 2015. Inpatient as well as outpatient (emergency department, outpatient clinic) data were considered.

The following sports were included in the evaluation: asphalt sports (inline skating and roller skating, skateboarding, longboarding), soccer, martial arts (boxing, judo, martial arts, kickboxing, wrestling, Thai boxing, taekwondo), contact team sports (basketball, ice hockey, football, handball, volleyball), cycling (BMX, downhill, bicycle, mountain bike, road bike), equestrian sports (horseback riding, rodeo, show jumping, vaulting), risk sports (parachuting, kite surfing, climbing, motocross, paragliding, surfing, windsurfing, wakeboarding), gymnastics/acrobatics (artistic gymnastics, children’s gymnastics, trampoline), and winter sports (skiing, snowboarding). The sports inline skating and roller skating, skateboarding, and longboarding were combined to form the superordinate sport of asphalt sports because they have similar accident mechanisms and riding speeds. Furthermore, ground conditions and protective equipment are similar. Soccer plays a very important role in Germany. Thus, due to the high proportion of people participating in soccer and the resulting high cases of injuries, it was studied separately from the other contact team sports. The individual risk sports were combined as described above, as the patients accepted a higher basic risk of injury when practicing these sports.

The main target variables recorded were sex (female = w, male = m), age at accident, type of sport, body region (head/face, shoulder, arm/elbow, hand/wrist, thorax/ribs, spine, pelvis/hip, leg/knee, ankle/foot), type of injury (avulsion fracture, bone bruise, commotio cerebri, contusio cerebri, contusio cordis, contusio spinalis, fracture, fissure, joint dislocation, cerebral hemorrhage, cartilage injury, compartment syndrome, dislocation, muscle injury, nerve injury, pneumothorax, tendon rupture, partial tendon rupture, serial fracture, serial dislocation), and exact localization of the injury.

Secondary outcome measures were insurance status (workers’ compensation or medical insurance), side of the injury (right, left), year of the accident, and, in the case of fractures, open or closed fracture (in the case of open fractures, indication of degree, e.g., second degree open), complication (yes, no), type of complication, and inpatient stay in days (added up in the case of more than one stay). Finally, the patients were divided into six age groups (0–16, 17–32, 33–48, 49–64, 65–80 > 80 years) and compared by gender.

Exclusion criteria for the presence of injury were nonstructural injuries such as sprains or contusions or just suspected diagnoses, unless the patients presented again to the outpatient clinic after external imaging was performed. In addition, injuries that did not clearly occur during sports were excluded, e.g., at the hut in a ski resort or injuries caused by the ski lift. Bicycling as a mode of transport was also excluded.

### Statistical Analysis

Data collected with Microsoft Excel were analyzed using IBM SPSS Statistics for Microsoft Windows, version 27.0 (Armonk, NY, USA: IBM Corp).

All *p*-values were two-sided, and *p*-values < 0.05 were considered statistically significant.

Absolute injury cases were counted using frequency tables. Mean value, standard deviations, and 95% confidence intervals of the variable “age at injury” were calculated using the descriptive statistic function of SPSS.

The aim of the statistical investigation was to test different variables for a possible correlation with the biological sex of the injured person. Of particular interest were the variables “age group”, “type of sport causing the injury”, and “injured body region”.

The actual observed frequencies were compared to the statistically calculated frequencies using Fisher’s exact test, and were later marked accordingly in the presentation of the results.

## 3. Results

The analysis of 20,567 inpatient and outpatient letters revealed 5455 patients with 7279 injuries. In total, 1475 of the patients were female and suffered 2035 injuries, and 3890 patients were male and suffered 5244 injuries.

**Figure 1:**
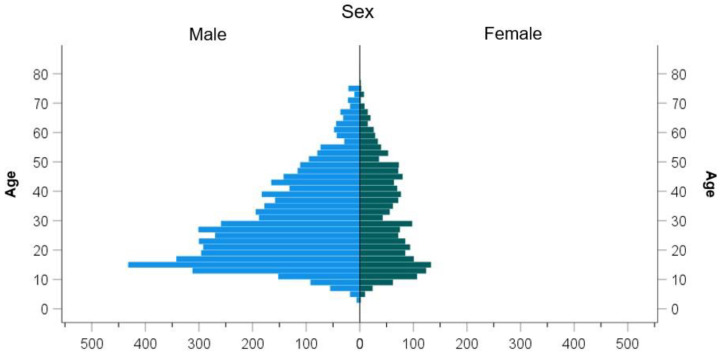
Distribution of injuries by gender and age.

Most of the injuries occurred in the age group of 17 to 32 in both females (w) and males (m) (31.1% of females, 41.1% of males). Female athletes ranged in age from 3 to 81 years, yielding a mean age of 30.90 years (SD = 16.261). Male athletes were between 3 and 83 years old, yielding a mean age of 28.90 years (SD = 14.769) (female athletes = female, male athletes = male, athletes = both male and female). Female athletes were thus on average two years older than male athletes when they suffered an injury (95% CI [1.188, 2.812], t (3409.169) = 4.83, *p* < 0.001) (Figure 1).

In relation to age, the following differences were found. Female patients ([M] 16; 95% CI; 13.46–18.54) and male patients ([M] 17; 95% CI; 14.85–19.15) who were injured while practicing gymnastics were on average the youngest compared to patients practicing other sports. Patients who were injured while practicing cycling had the highest mean age ([M] 48; 95% CI; 41.08–54.92). Patients exercising horseback riding ([M] 43; 95% CI; 40.58–45.42) or practicing winter sports ([M] 43; 95% CI; 41.84–44.16) (see Table 1) were the oldest when they were injured.

When considering all sports, soccer injuries were the most common (n = 3057), and the proportion of male patients was high (n = 2872). Most injuries in female athletes occurred in equestrian sports (n = 704). Overall, 72.0% of injuries were attributable to male patients and 28.0% to female patients.

### 3.1. Age Groups

There were no significant relationships for asphalt (*p* = 0.179), contact (*p* = 0.074), high-risk (*p* = 0.117), winter (*p* = 0.101), and gymnastics/acrobatics (*p* = 0.101) sports. Female athletes were injured more frequently than expected in martial arts and soccer when they were in the age group 0–16 (*p* = 0.005 and *p* = 0.001, respectively). The highest risk of injury while playing soccer was for men in the age group 33–48 (*p* = 0.001). Female cyclists were more likely expected to be injured in the age group 65–80 (*p* < 0.001) and over 80 (*p* = 0.001). Female and male equestrians showed significant deviations from expected injury rates in all age groups. Female equestrians showed more injuries than expected at ages 0–16 (*p* < 0.001) and 17–32 (*p* < 0.001), while male equestrians at ages 33–48 (*p* < 0.001), 49–64 (*p* < 0.001), and 65–80 (*p* < 0.001) exceeded expectations for injury rates. Comparing all sports, injuries in female athletes in the 33–48 (*p* = 0.001) and 49–64 (*p* < 0.001) age groups stood out, while athletes between 17 and 32 (*p* < 0.001) had significantly more injuries than expected (Table 2).

### 3.2. Type of Sport

A statistical test was performed to determine whether there was a correlation between the sex of the athletes and the sport in which the injury occurred. The test was based on the assumption that the respective sexes tended to participate in certain sports and thus were injured more frequently than the other sex or generally more frequently than expected.

**Figure 2:**
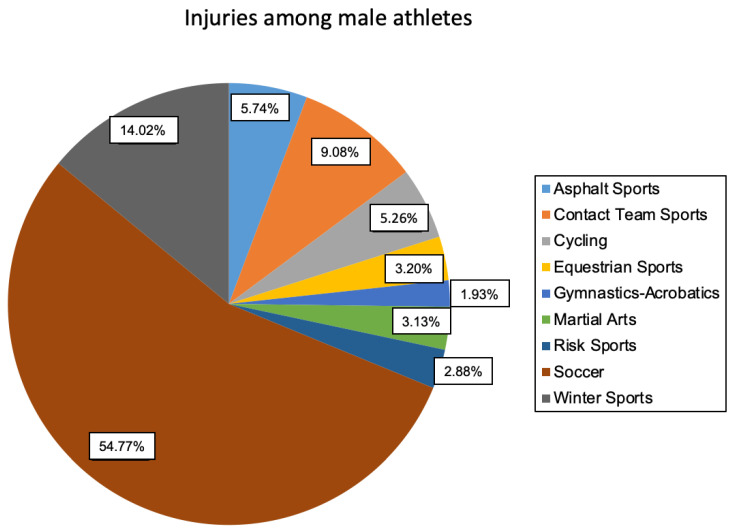
Distribution of injuries among the sports studied (male athletes).

**Figure 3:**
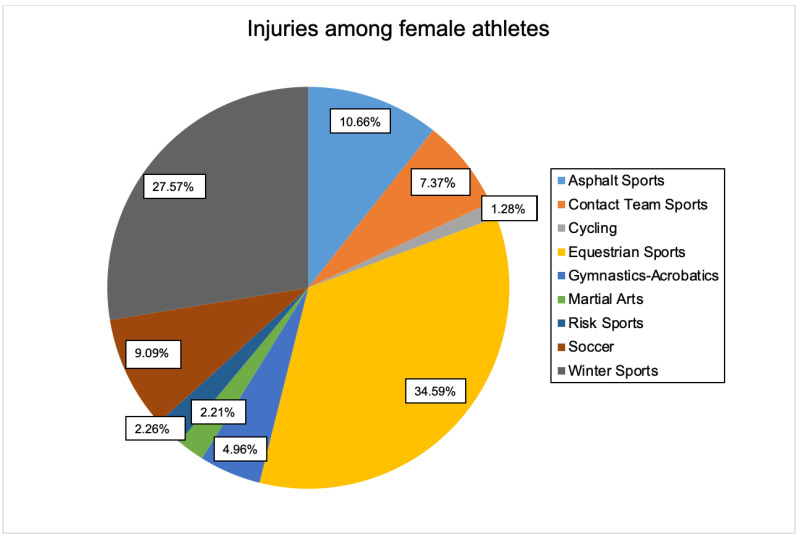
Distribution of injuries among the sports studied (female athletes).

Statistical analysis confirmed the presumption of a relationship (*p* < 0.001, w) (see Figure 2 and Figure 3).

The sports that had a statistically significant effect on the correlation between injury and gender were recorded as variable levels. Asphalt sports (*p* < 0.001), cycling (*p* < 0.001), equestrian sports (*p* < 0.001), gymnastics/acrobatics (*p* < 0.001), winter sports (*p* < 0.001), and soccer (*p* < 0.001) proved to be influential in this regard.

Furthermore, the “direction” of the relationship could subsequently be examined in more detail. More female athletes than expected were injured in asphalt sports (w = 217, “w” _”μ” = 144.8), equestrian sports (w = 704, “w” _”μ” = 243.8), gymnastics/acrobatics (w = 101, “w” _”μ” = 56.5), and winter sports (w = 561, “w” _”μ” = 362.3). However, they did not show statistical significance. In contrast, significantly more athletes than expected were injured in cycling (m = 276, “m” _”μ” = 217.6) and soccer (m = 2872, “m” _”μ” = 2202.4).

### 3.3. Body Region

In the following popular sports, dependencies of the injured body region on biological sex have been found: asphalt sports (*p* < 0.001), contact sports (*p* < 0.001, V = 0.21), cycling (*p* = 0.004), equestrian sports (*p* < 0.001), winter sports (*p* < 0.001), gymnastics/acrobatics (*p* = 0.007), and soccer (*p* = 0.009).

Bonferroni correction of the standardized residuals of the variable “region of injury” allowed us to define body regions for four of these seven sports that significantly influenced injury dependence (Table 3).

Among male athletes, shoulder injuries in asphalt sports (*p* < 0.001) and winter sports (*p* < 0.001) contributed to the significant correlation of injured body region and gender. Arm and elbow injuries were more common than expected in female cyclists (*p* > 0.001) and additionally more common in female equestrians than male equestrians (*p* = 0.002). In contrast, in winter sports, arm and elbow injuries were unexpectedly more common in men (*p* = 0.001), while women had clustered injuries to the pelvis/hip (*p* < 0.001) and leg/knee (*p* < 0.001) regions. Injuries in women practicing asphalt sports occurred strikingly more frequently in the spine region (*p* < 0.001). Finally, equestrians were found to be significantly more likely (*p* < 0.001) to be injured to the pelvis and hip regions than expected.

#### Types of Injury

In the next step, the most frequent injury types (e.g., fracture, tendon rupture, etc.) were selected for each body region that significantly influenced the correlation between body region and gender. The most common exact locations (e.g., radial head, upper ankle) were then identified for these injury types. Athletes were most likely to show clavicle fractures and shoulder joint dislocations in sports in which injuries to the shoulder body region significantly contributed to the gender difference (asphalt and winter sports).

The injuries of the arm/elbow body region that occurred unexpectedly often in female cyclists were solely fractures (n = 10). The most common were radial head fractures (n = 4) and fractures of the distal humerus (n = 2).

Female equestrians also suffered fractures most frequently in the arm/elbow body region (n = 120), most of which occurred in the distal (n = 29) and proximal humerus (n = 29). After fractures, dislocations (n = 11) appeared as the next most common injury type, most of which were dislocations of the elbow joint (n = 8).

Male winter sports athletes showed the most frequent fractures (n = 122) among injuries to the arm/elbow body region, with fractures of the proximal humerus (n = 40) and humeral head (n = 39) standing out in particular.

Among female winter sports athletes, the leg/knee region showed predominantly tibial plateau fractures (n = 69) and anterior cruciate ligament tears (n = 52). In the pelvis/hip body region, fractures (n = 28) occurred most frequently, especially fractures of the pubic bone (n = 7) and sacrum (n = 6). In the association between sex and injured body regions in female asphalt athletes, spine injuries were predominant.

Fractures (n = 14) emerged as the most common injury type, most of which were due to fractures of the thoracic (n = 6) and lumbar (n = 6) spine.

Although fractures were normally the most common type of injury, this was not the case for injuries to the pelvis/hip region in equestrians. Here, joint dislocations (n = 18), or, more precisely, symphysis dislocations (n = 15), were the most common injury. Fractures (n = 17) presented as the second most frequent type of injury, and were mainly fractures of the sacrum (n = 7).

## 4. Discussion

A total of 7279 injuries were included over a 16-year period. Sports with similar characteristics (behavior, equipment, contact vs. individual sport, ground condition, etc.) were grouped. Only soccer was viewed separately due to its high status in German sports culture.

The results obtained are discussed using soccer as an example, highlighting how the differences between the two genders are presented.

With more than seven million club members (inventory survey of female and male members of the German Olympic Sports Confederation) (2021), it is not surprising that most injuries in German club sports occur while playing soccer. This can also be confirmed by the results of this work. Soccer accounted for the highest number of injuries in the present population (N = 3057) with simultaneously low patient age (w = 20 years, m = 25 years). According to prevailing literature, the distribution of injury prevalence in male athletes in different sports has remained constant over the years. Thus, athletes are injured most frequently in the classic ball sports of soccer, handball, volleyball, and basketball. Furthermore, an interesting change has been found in female athletes over the past few decades. While most injuries occurred in handball, there has been a consistent decrease in injuries caused by “classic” sports such as gymnastics, acrobatics, and volleyball, but again a significant increase in soccer injuries [9]. It can be speculated that there has also been a change in the types of injuries among female athletes that coincide with higher frequency of practicing injury-causing sports. After all, in contrast to gymnastics, acrobatics, and volleyball, handball and soccer are sports that frequently involve injuries caused by direct contact with opponents (e.g., duels). In the 2018/2019 season, 51.4% of soccer injuries and 49.7% of handball injuries [10] in each of the two highest men’s leagues could be linked to opponent contact. It should again be noted that only the men’s leagues are included in the accident monitoring of the “Verwaltungsberufsgenossenschaft (VBG)” (professional association/workers compensation) in an annual report. A comparable report for female leagues is missing. The collected data of the present work show an absolute number in contact and team sports of w = 150 and m = 476, with an average age of w = 24 years and m = 27 years. In our view, the proportion of female athletes is too high to not establish sex-specific prevention and therapy.

There are already well-evaluated prevention programs to significantly reduce the injury rate, e.g., in soccer. For the prevention program FIFA 11, however, the studied sports collective is mainly male. No monocausality can be derived at this point, but it must be stated that, based on the available data, it would be practical to develop more prevention programs for female athletes. This underlines the importance of preventative measures related to biological sex as well as gender-specific therapy options.

The retrospective collection of data is the main limitation of this study. The facts were mainly extracted from the medical history of the respective stationary and ambulance letters. For most sports, the reference of a specific sport is unambiguous; however, there are sports such as “cycling” that are also used as a mode of transportation. We tried to filter these out and not include them in the statistics, but we could not always check this for 100% accuracy.

Furthermore, it can be stated that, due to the retrospective evaluation, we were not able to record all aspects of content. Some questions remained unanswered, e.g., if warm-up training was being performed, or if protective equipment was used. However, this was not the aim of this work. Rather, our goal was to list the differences in sports injuries in a gender comparison in order to emphasize the importance of gender-specific training, prevention programs, and therapy concepts. In childhood, adolescence, and adulthood, anatomical differences as well as different sports behavior of both sexes resulted in different patterns and extents of injuries. The above results show the differences between the male and female sex with regard to sport-specific injuries. The results emphasize sex-specific approaches to both therapy and prevention. Sports participation is a crucial health-promoting factor and should be supported by sex-appropriate prevention programs and adapted therapy plans. The present work offers a basis in pointing out the sex differences in injuries, both in the specific type of sport and in the analysis of the affected body regions.

## 5. Conclusions

The results of this study show that there are significant differences in both localizations and types of sports injuries between genders in different age groups. In our opinion, the prevention programs and treatment approaches that exist today do not adequately reflect this diversity. This retrospective epidemiological study is intended to provide a basis for additional specific work in this area to meet the sporting needs of both sexes.

## Figures and Tables

**Table 1:** Age of female (w) and male (m) patients, mean (M) with 95% confidence interval (95% CI), standard deviation (SD), and number of injuries (N).

Sports	Sex	N	Age	Age	Age	Age
			Minimum	Maximum	M (95% CI)	SD
Asphalt Sports	f	217	5	57	27 (25.14–28.86)	14
	m	301	6	75	26 (24.31–27.69)	15
Soccer	f	185	7	67	20 (18.70–21.30)	9
	m	2872	3	76	25 (24.60–25.40)	11
Martial Arts	f	45	10	45	23 (19.79–26.21)	11
	m	164	5	58	25 (23.32–26.68)	11
Contact Team Sports	f	150	9	63	24 (22.08–25.92)	12
	m	476	6	70	27 (25.92–28.08)	12
Cycling	f	26	15	81	48 (41.08–54.92)	18
	m	276	9	75	35 (33.11–36.89)	16
Equestrian Sports	f	704	5	74	28 (26.97–29.03)	14
	m	168	8	75	43 (40.58–45.42)	16
Risk Sports	f	46	7	53	34 (30.53–37.47)	12
	m	151	6	69	38 (35.61–40.39)	15
Gymnastics-Acrobatics	f	101	3	78	16 (13.46–18.54)	13
	m	101	3	59	17 (14.85–19.15)	11
Winter Sports	f	561	5	77	43 (41.76–44.24)	15
	m	735	3	83	43 (41.84–44.16)	16

**Table 2:** Bonferroni-corrected significant sex differences split by sport and age group, expected value female/male (*w*/*m*).

Sports	0–16	17–32	33–48	49–64	65–80	> 80
All	-	m > m_μ_	f > f_μ_	f > f_μ_	-	-
	-	m > f	-	-	-	-
	-	*p* < 0.001	*p* = 0.001	*p* < 0.001	-	-
Martial Arts	f > f_μ_	-	-	-	-	-
	-	-	-	-	-	-
	*p* = 0.005	-	-	-	-	-
Soccer	f > f_μ_	-	m > m_μ_	-	-	-
	-	-	m > f	-	-	-
	*p* = 0.001	-	*p* = 0.001	-	-	-
Cycling	-	-	-	-	f > f_μ_	f > f_μ_
	-	-	-	-	-	f > m
	-	-	-	-	*p* < 0.001	*p =* 0.001
Equestrian Sports	f > f_μ_	f > f_μ_	m > m_μ_	m > m_μ_	m > m_μ_	-
	f > m	f > m	-	-	m > f	-
	*p* < 0.001	*p* < 0.001	*p* < 0.001	*p* < 0.001	*p* < 0.001	-

**Table 3:** Dependence on body region, gender and type of sport.

Sports	Shoulder	Arm/Elbow	Spine	Pelvis/Hip	Leg/Knee
Asphalt Sports	m > m_μ_	-	f > m	-	-
	m > f	-	f > f_μ_	-	-
	*p* < 0.001	-	*p* < 0.001	-	-
Cycling	-	f > f_μ_	-	-	-
	-	-	-	-	-
	-	*p* < 0.001	-	-	-
Equestrian Sports	-	f > f_μ_	-	m > m_μ_	-
	-	f > m	-	-	-
	-	*p* = 0.002	-	*p* < 0.001	-
Winter Sports	m > m_μ_	m > m_μ_	-	f > f_μ_	f > f_μ_
	m > f	m > f	-	f > m	f > m
	*p* < 0.001	*p* = 0.001	-	*p* < 0.001	*p* < 0.001
